# Revision Trabeculectomy: Pearls and Pitfalls

**DOI:** 10.5005/jp-journals-10008-1120

**Published:** 2012-10-16

**Authors:** Michael Coote, Jonathan Crowston

**Affiliations:** Glaucoma Facility, Royal Victorian Eye and Ear Hospital and Centre for Eye Research, Melbourne, Australia; Professor and Head, Glaucoma Research Unit, Royal Victorian Eye and Ear Hospital and Centre for Eye Research, Melbourne, Australia

**Keywords:** Bleb revision, Bleb failure, Trabeculectomy, Glaucoma surgery, Revision trabeculectomy.

## Abstract

Revision trabeculectomy is used to describe any surgical intervention subsequent to an existing trabeculectomy. Mostly, it is used to describe resurgery for failure of trabeculectomy, as defined by inadequate pressure control. Revision may also be performed for unsafe, uncomfortable or leaking blebs.

Mostly bleb failure occurs within the subconjunctival space, although the flap and ostium may be involved or causative. Clear surgical principles, meticulous surgical technique and scrupulous postoperative care are key to successful revision surgery. This review is an attempt to elucidate the technique of bleb revision for bleb failure.

**How to cite this article:** Coote M, Crowston J. Revision Trabeculectomy: Pearls and Pitfalls. J Current Glau Prac 2012;6(3):131-138.

## INTRODUCTION

The term ‘Revision trabeculectomy’ is used to describe any subsequent surgical intervention to an existing trabeculectomy. Mostly, it is used to describe resurgery for failure of trabeculectomy, as defined by inadequate pressure control. Revision may also be performed for unsafe, uncomfortable or leaking blebs.

In this chapter, we will focus on revision for bleb failure. Mostly bleb failure occurs within the subconjunctival space, although the flap and ostium may be involved or causative.

Revision trabeculectomy for bleb failure may be performed via limbal or fornix incision. The posterior (fornix) approach appears to offer improved outcomes with fewer complications. Revision using needling is not covered in this chapter. It has a legitimate place in the management of dysfunctional blebs, although the longer term success rates are less than for formal revision.

In most circumstances of failed trabeculectomy revision is the preferred operation. In some circumstances, a revision may be very unlikely to offer successful and safe filtration over an acceptable duration and thus moving to other options is appropriate. Many clinicians have developed strategies and treatment algorithms based on their own, and institutions, experience. In these circumstances, cyclo-destructive procedures or tube implantation may be second line rather than revision.

Revision trabeculectomy is an almost completely extraocular procedure, which can be repeated, and it is most often tolerated well by the eye and the patient. It offers the potential for the same amount of postoperative manipulation and adjustment as a trabeculectomy does, which is in contrast to cyclodestructive procedures or tube implants. Revision trabeculectomy offers the possibility of low and predictable pressures.

Revision trabeculectomy has historically been considered as a technically difficult and low success rate procedure. Clear surgical principles, meticulous surgical technique and scrupulous postoperative care are key to successful revision surgery. With attention to these issues, high success rates can be achieved.^1-7^

## PREOPERATIVE ASSESSMENT FOR REVISION TRABECULECTOMY

### Type of Glaucoma is Relevant to the Risk of Failure

Uveitis, ICE syndrome and trauma are particularly high risk for failure of primary filtration surgery and are high risk for failure of revision as well. These eyes may require a different surgical strategy to obtain a satisfactory outcome.

### Time since the Original Operation is an Important Variable

Gradual failure in the subconjunctival space often creates delineated spaces with pseudoendothelialization, which makes reoperation more straightforward and the areas of obstruction more clearly defined. Earlier failure is associated with more diffuse scarring and suggests a higher risk patient. Early failure will require a substantial intraoperative antimetabolite as well as intensive postoperative care. The exception to this is where the primary operation has failed to function at any time - in which case primary sclerostomy failure is more likely. This underscores the value of gonioscopy in pre- and postoperative management of trabeculectomy surgery.

### Identifying the Target Intraocular Pressure

If a low target pressure is not required, there is poor capacity for sight in the eye, or outflow surgery is not likely to be achieved, then cyclodestructive procedures (either trans-scleral or endoscopic) may be more suitable interventions. A low target intraocular pressure in a seeing eye will generally require a functioning trabeculectomy. In general, if the eye has required an operation to control the pressure, it will need one to continue to function.

### Surgical Method of Previous Procedure

It is useful for the revision surgeon to understand the original wound site, concentration and extent of antimetabolite used, the size of flap and closure, the size of sclerostomy and the presence or absence of peripheral iridectomy in the primary procedure. Previous operative notes should contain this, but clues may be obtained from the postoperative course. For example, if the eye developed prolonged hypotony and then subsequently failed, it may be that insufficient aqueous flow was never established in order to provide the bleb space. In this circumstance, the use of retained viscoelastic, perioperative atropinization and restriction of patient activity postoperatively will help to reduce the chance of this reoccurring at revision.

### Assessing the Conjunctiva and Tenon’s

Conjunctiva may be indifferent to scarring of Tenon’s or the two may be joined in a conjunctival encysted bleb (see below). Tenon’s varies in its development, attachments and response to filtration surgery. At the slit lamp, it is not possible to accurately assess Tenon’s adhesion and these areas may be quite separate to conjunctival adhesions. Mobility of conjunctiva, even if Tenon’s is adherent to sclera, will make the development of a bleb substantially easier. If possible, draw out areas of conjunctival adhesion preoperatively, which will be confirmed intraoperatively.

### Where did the Previous Operation Fail?

Revision of trabeculectomy is made more straightforward by understanding the site of the failure and what sort of surgery was undertaken initially. The most common site of failure of trabeculectomy is in the subconjunctival space, usually with an open sclerostomy and a patent peripheral iridectomy. If this is not the case then opening of the sclerostomy or removal of obstructive material, which includes iris, vitreous or lens matter, may be required. Although the site of failure will be ultimately defined at the time of surgery, good preoperative workup will reduce the likelihood of surprise.

### Bleb Failure Type

**Figs 1A and B:**
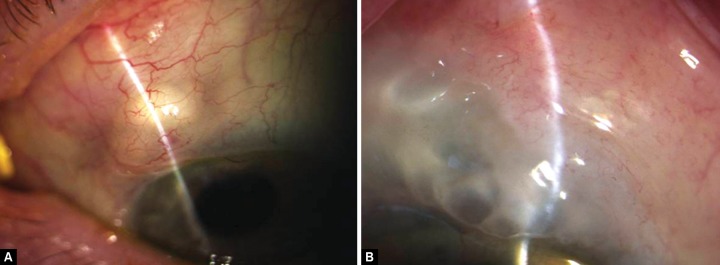
(A) Tenon’s encystment, (B) conjunctival encystment

The failure of permeability of the bleb may occur at different levels. In the surgical reconstruction of the bleb, the preoperative identification of where the failure has occurred is helpful. In essence, the most significant difference is between encystment which is restricted to the sub-conjunctival tissue (Tenon’s encystment) and encystment involving all layers (Conjunctival encystment). In the latter consideration will need to be given to excising all of the bleb and advancing the conjunctiva (Fig. 1).

## SURGICAL STRATEGY FOR POSTERIOR REVISION OF TRABECULECTOMY

### Access

**Fig. 2:**
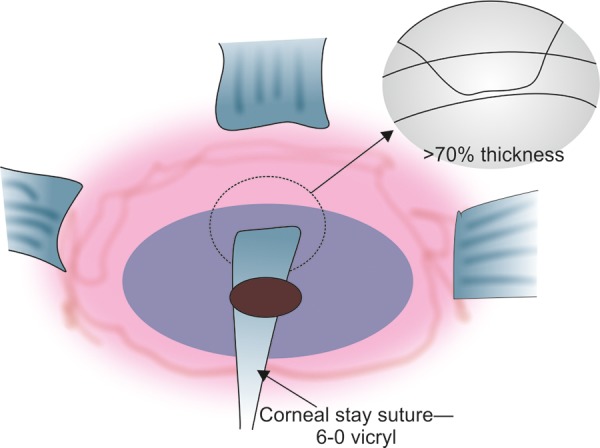
Corneal stay suture for proper exposure

As the initial incision in a posterior revision will be close to the fornix, the eye will need to be rotated inferiorly as far as possible. The most efficient strategy for this is to use a 6-0 braided suture passed through greater than 70% of corneal thickness, 1 mm from the limbus, adjacent to the previous trabeculectomy (Fig. 2).

The eye is then pulled directly down in line with the previous trabeculectomy and the suture is clamped to the drape. Sufficient anesthetic is required in order to relax the superior rectus, if there is still significant superior rectus activity then further sub-Tenon’s infusion of anesthetic is appropriate.

It may not be necessary to gain access to the entire area of the bleb. For example, if the original operation is superonasal, then it may be more reasonable to extend the bleb superotemporally and thus access would be more centered around the temporal aspect of the bleb. The initial incision is made as far as practical away from the limbus and the operative field and wide enough to allow full access to the area of failure. The initial incision should be down to sclera, being careful to identify muscle.

In general, the new bleb will be fashioned in such a way to extend between superior rectus and either lateral or medial rectus. Identification of intermuscular septum, division and opening of the posterior space is required at this stage.

**Fig. 3:**
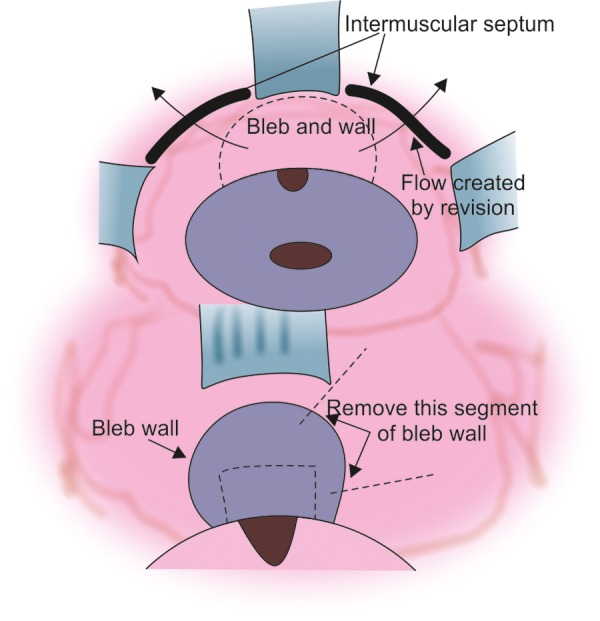
Flow of aqueous following bleb revision

**Fig. 4:**
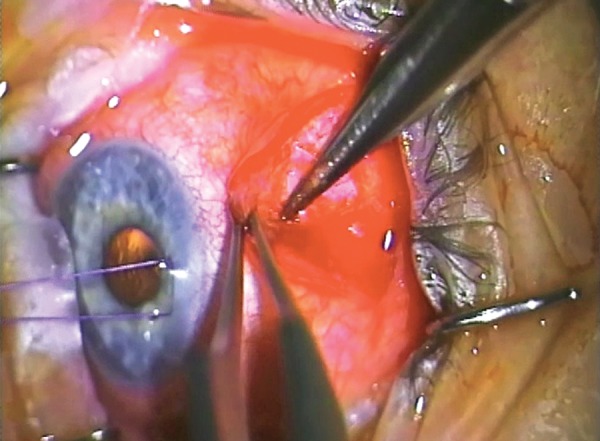
Advancing forward in the subconjunctival space against the sclera until the fibrosed encapsulation is encountered

**Fig. 5:**
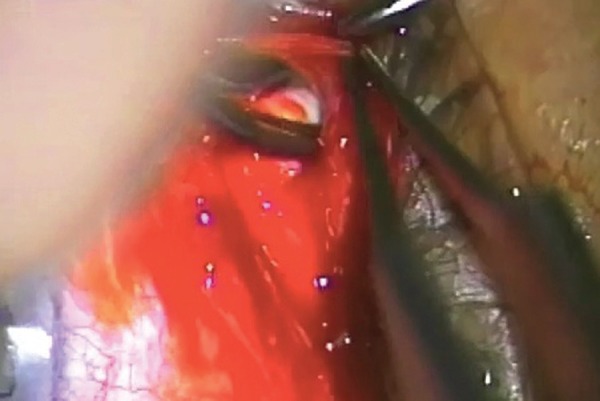
Posterior dissection through the intermuscular septum into the intermuscular space

### Dissection of Bleb Wall

Advancing at the level of the sclera toward the limbus, there may be a gradual or relatively abrupt development of impervious scar. This is the usual reason for failure of a trabeculectomy. The absence of such a scar suggests primary sclerostomy or flap failure or internal ostial failure (Figs 3 to 10).

It is usually possible to demarcate the bleb/capsule wall that will need to be removed. Not all of the bleb/capsule wall needs removal for a successful revision, but at least one half of the wall directed toward where the bleb will be developed will need to be excised. Remove sufficient bleb wall to make reclosure of the capsule impossible (See Fig. 3).

Blebs that are many years old develop pseudo-endothelialized remnants which have a glistening (almost cartilaginous) appearance to them. These components need to be removed, at least on their scleral surface, with the attempt to pare back and remove all of the scar tissue to the original scleral level. It is not necessary to remove all of the roof bleb elements. If the central part of the bleb is thin and cystic, then care needs to be exercized in the dissection lest there is inadvertent perforation of the roof.

**Fig. 6:**
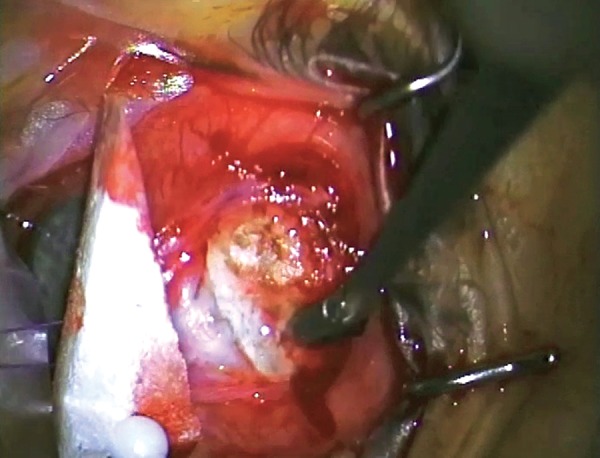
Advancing until there is free flow of aqueous

**Fig. 7:**
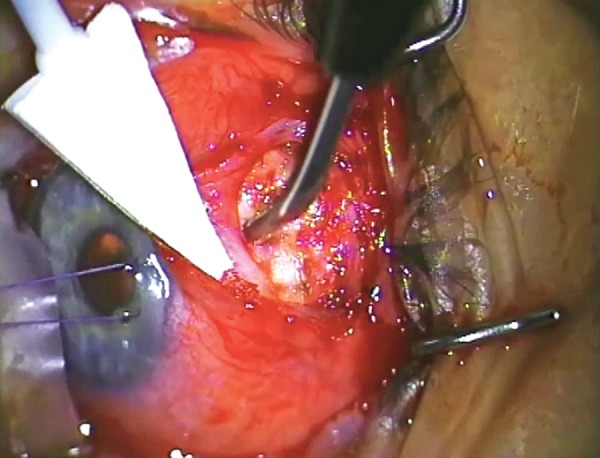
Vigorous diathermy of the base of the bleb which is often very vascular

**Fig. 8:**
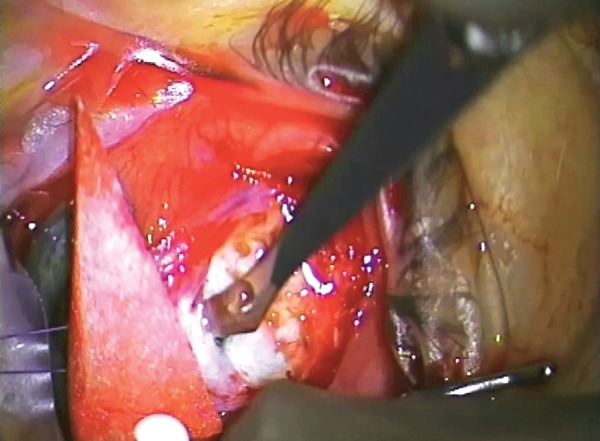
Lifting the flap or remnant to gain unimpeded access to the anterior chamber

**Fig. 9:**
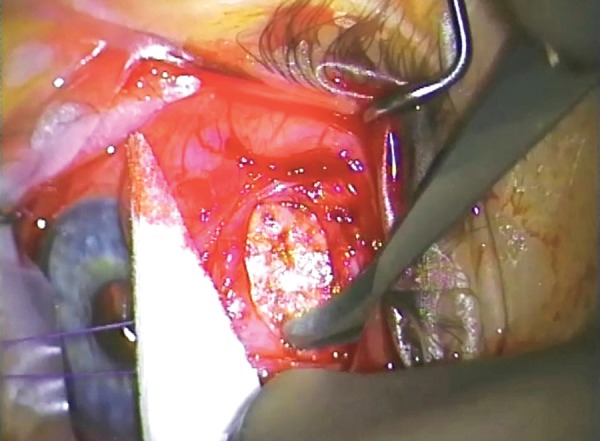
Sharp dissection of the more organized fibrous bleb anteriorly using a #67 ‘Beaver’ blade or similar

**Fig. 10:**
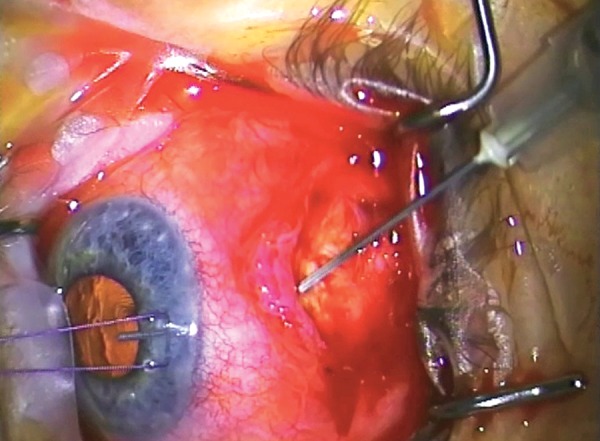
Filling the anterior chamber with a viscoelastic device (in this case ‘Healon’)

### Identification and Opening of the Scleral Flap

In revision surgery, one use of the flap is to protect the roof of the bleb, if it is thin. It is usually not intended to offer any resistance to outflow or distribute aqueous as it does in the primary procedure. Resistance is provided in the subconjunctival space which has already developed scar from previous surgery. Distribution occurs due to the bleb space being more mature and open.

If the risk of postoperative hypotony is judged as high, then placement of a suture(s) to restrict outflow may help, although regaining watertight closure is usually technically difficult.

Identification of the original flap may also be difficult, as it may have become completely bound down and almost invisible due to condensation of scar tissue over the flap. Sometimes the flap is thin and incomplete, even up to virtual dissolving of the flap with the development of a full thickness sclerostomy. In either case, the aim is to provide a sufficiently loose and open flap, such that no realistic impediment to aqueous outflow occurs at this point.

### Sclerostomy

Trabeculectomy can fail at the sclerostomy site, such as an imperforate Descemet’s membrane or inadequate sclerostomy size which has closed with scar. A sclerostomy placed too anterior under a larger flap may have effective closure with contraction of scar over the top of the flap. The key dimension in trabeculectomy sclerostomies and flaps is the amount of overlap of the flap to the sclerostomy –not the size of each component. If there is significant overlap between the flap and the sclerostomy, then the sclerostomy will need to be enlarged.

Although sclerostomies, in theory, need only to be 50 to 100 microns in order to support unobstructed outflow, in reality they need to be sufficiently larger than this as sclerostomies of these dimensions will close with the development of scar.

It is usual to leave a viscoelastic device (VED) in the eye after revision. It is, thus, imperative that the outflow from the eye is unimpeded otherwise the eye may develop very high IOP in the postoperative period. Remember that the VED will tend to close any flap valve that may exist at the internal ostium.

### Iridectomy

It is imperative to identify the internal structures and be reassured that no obstruction to outflow is occurring at the internal ostium. This includes iris, vitreous or lens material that may be present. If the iris is imperforate, then a full thickness iridectomy is mandatory. If vitreous is identified, then a vitrectomy through the sclerostomy/iridectomy will be required using a formal aspiration and irrigation cutting technique. The aim is clear sufficient vitreous to prevent forward movement again, especially in the hypotonous postoperative period.

### Recreation of the Bleb

Once access to the anterior chamber has been established, the next stage of the procedure is development of a bleb.

Identify where the Bleb will be

Having fully dissected subconjunctival space in order to gain access to the internal ostium, it may now be necessary to widen this area, diathermy it and remove any further scar or endothelialized elements present. A posterior opening behind the intermuscular septum and dissecting circumferentially may be useful. If approaching the bleb from only one side, then substantial flow will need to occur both posteriorly and circumferentially or, if approaching the bleb immediately posteriorly, then it may be necessary to dissect behind both intermuscular spaces beside the superior rectus.

The goal at this stage is to establish the physical dimensions of the bleb, which will be fortified chemically with antimetabolite application.

Diathermy

The effect of diathermy is not merely to prevent bleeding: Diathermy creates dead tissue (which excites an inflammatory action) but will also denude areas of (primed) fibroblasts. In addition, diathermy will augment the use of mitomycin and reduce inflammatory exudation, if performed satisfactorily.

Diathermy needs to be extensive in areas where there has been bleb wall formation and where there has been preexisting condensation of fibrous tissues. Once diathermy has been performed, it may be possible to excise more of the scar tissue and rediathermy underneath.

**Fig. 11A:**
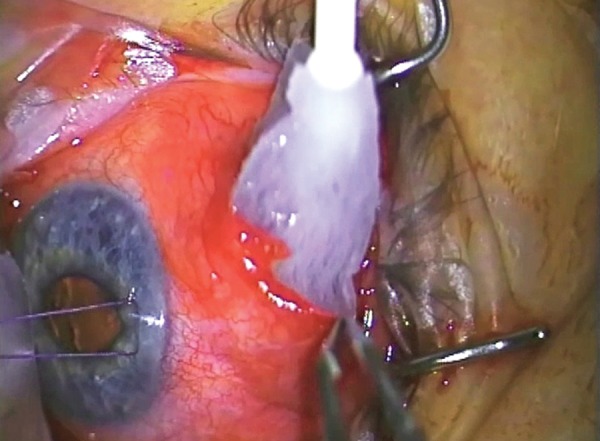
Placing a triangular sponge with 0.4 mg/ml mitomycin C over the area of the flap and far back to the intermuscular space (with sufficient viscoelastic device in AC to prevent mitomycin C entrance)

**Fig. 11B:**
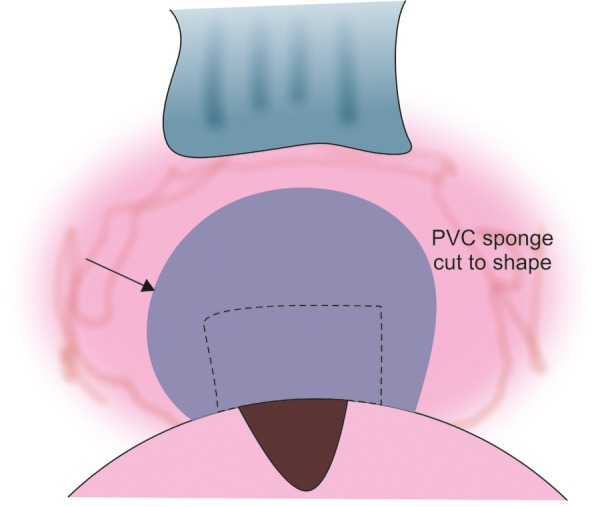
Antimetabolite application using sponges

Application of Antimetabolite

All revision trabeculectomies will require the application of mitomycin-C (MMC) or a similar long-acting antifibrotic. >MMC is applied using a sponge cut to shape to fill the space created after the area has been dissected and diathermied (we use a polyvinyl chloride instrument wipe). One end of the sponge should come to rest essentially at the sclerostomy and the other end should extend through the intermuscular space (Figs 11A and B).

Prior to the application of MMC, the anterior chamber is filled with viscoelastic so that there positive pressure in the anterior chamber and no aspirational pressure on the MMC.

In the revision operation, the concentration of MMC (usually between 0.2 mg/ml and 0.4 mg/ml), the area over which it is applied and the length of application (usually 1-4 minutes) all need adjustment by the surgeon for each case. The factors that are involved in the decision are the density and thickness of material and the volume of distribution. Thinner sponges may require resoaking and reapplication over the time of application, whereas a larger volume of sponge will provide a larger reservoir for the drug to equilibrate.

Vascular tissue tends to absorb the drug with less effect, and conversely the application of diathermy tends to augment the MMC. This is also true of drying of the tissue and there may be quite pronounced augmentation in the effect of MMC, if the sclera is quite dry (and transparent) before application.

The roof of the bleb and the subconjunctival tissue needs adequate MMC application as well.

### Closure

Emptying of Viscoelastic

Prior to formal closure of the wound, it is vital to confirm the viscoelastic, which is left in the eye, can empty easily into the subconjunctival space. This is usually performed by pressing on the eye inferiorly and watching the viscoelastic empty. Remember there is a tendency for a valve effect and for viscoelastic to get trapped in the anterior chamber so the surgeon needs to be very sure that it can egress easily.

Closing Conjunctiva

Conjunctiva is closed in a single layer using a braided 8-0 absorbable suture, although other suture types are possible. This is usually combined with a blood vessel or taper needle, which creates smaller punctures. In general, small bites on the anterior ridge and larger bites posteriorly will create less tendency for the incision to move limbally. It is not necessary to pick up conjunctiva posteriorly at every bite as the likelihood of leak from this area is very low, although physical closure of the wound with Tenon’s is important.

Immediate Postoperative Management

Using a paracentesis port, make sure the anterior chamber is filled. Make sure that fluid injected into the anterior chamber can fill the bleb. If there is a significant concern of postoperative hypotony, then further viscoelastic through a side port may be added.

Dexamethasone injected directly into the bleb and the subconjunctival space or trapped between the conjunctiva and the Tenon’s. In general, no antibiotic is required to be given subconjunctivally.

The eye is padded with antibiotic ointment. If there is a significant risk of postoperative hypotony, then atropine is added.

## PEARLS

### Preoperative Assessment

All patients who are undergoing revision trabeculectomy need gonioscopy and assessment of conjunctival mobility on the slit lamp. This will help to plan what is an appropriate procedure. If there has been primary sclerostomy failure with iris incarceration, it may be that the subconjunctival space has never been tested and therefore extensive dissection and wide application MMC will not be necessary. Conversely, if there is extensive conjunctival adhesion in a high risk patient, then an alternative surgical strategy may be appropriate.

### Access

Using a corneal stay suture to pull the eye as far as you can down and start a long way back. If there is still superior rectus activity, then infuse anesthetic around the superior rectus at the time. Access all the way to the fornix is important and it may be necessary to even do a lateral tarsorrhaphy in order to get access. Adequate anesthesia is vital and this is not a procedure to be attempted under topical anesthesia or while on anticoagulants or antiplatelet agents, unless the risk of stopping these agents is judged as unacceptable.

### Subconjunctival Space

From the initial incision, it is important to get down to sclera. Superior rectus is likely to be within the surgical field and early identification is helpful–cutting the superior rectus is clearly unhelpful. The superior rectus has radial blood vessels but it is useful to measure 5 to 6 mm in the limbus superiorly to identify its likely insertion point.

The intermuscular septum requires division and it is critical to explore in between superior rectus and lateral or medial rectus, or both. At this point, it is imperative to stay on the sclera and to fashion a pocket posteriorly.

### Flap

Do not try to refashion the scleral flap; just lift it to keep it open. The flap is no longer useful, in fact is most often an impediment. If there is some concern that the flap will impede outflow, then amputate the posterior aspect of it in order to be clear that it will not close due to contracting pressure above from scar. If there is a high risk of postoperative hypotony with significant consequences, then some form of releasable closure of the flap may be possible.

### Anterior Chamber and Flap

Watch for trauma and inflammation of vitreous in the anterior chamber, and watch an unusually shallow anterior chamber as it may indicate aqueous misdirection. You may need to punch the sclera to keep it open using a corneoscleral punch. Remember that diathermy around the edge of the sclerostomy will tend to reduce the likelihood of both bleeding and closure, and it may also be necessary to diathermy the root of the iris to reduce bleeding.

### Make a Bleb

Strategically, the surgeon is creating a bleb. The sponge needs to be laid exactly, where the bleb should be. If there has not been application of mitomycin in a particular area, then it is unlikely that a bleb will form there so it is imperative that mitomycin is laid carefully.

### Closing the Wound

A posteriorly situated wound does not need to be overly watertight. Further the wound is from the sclerostomy, the less likely there will be significant leak. One of the advantages of a posterior revision is that wound closure is not as important. The other significant advantage is that it traverses the subconjunctival scar form the outside in, thus allowing much clearer delineation of the structures.

### Postoperative Management

This procedure requires intensive postoperative steroids and with or without 5-FU. Aqueous suppressants may be useful, if the IOP rises above 15 mm Hg or the bleb becomes ischemic.

### Pitfalls

Insufficient Bleb

The most common pitfall is not to create sufficient area for the bleb. The solution is a larger sponge and subconjunctival dissection. The role of antimetabolites is to prevent subconjunctival scarring as well as to maintain the patency of the sclerostomy. Laying antimetabolites over the whole anticipated bleb area is vital for the success of the procedure.

Sclerostomy not Adequately Open

Another common area of failure is not opening the sclerostomy adequately. There needs to be free flow through the sclerostomy as the outflow resistance in this procedure will lie on the subconjunctival space much earlier than in the primary trabeculectomy. This will initially produce very low pressures but the subconjunctival space has already been identified is capable of producing resistance and will already have substantial resistance. If the hypotony is unacceptable, it is possible to mitigate this with a suture closure of the bleb.

Excess Antimetabolite

If there has been primary sclerostomy failure early in the postoperative phase, then the subconjunctival space has not been ‘tested’. In this circumstance, the tissue response is unknown and a second dose may result in an excessive tissue response with large areas of the pallor.

Not Resecting the Pale Leaking Bleb

If the center of the bleb is very pale or ischemic, then avoid putting further mitomycin in this area. It is likely that a sclerostomy is not the source of the problem and failure has occurred in the wall of the bleb. Thus, application of Mitomycin does not extend all the way to the sclerostomy and only to the wall of the bleb. Consider removal of the ischemic area with anterior suturing but in concert with posterior revision. This will then, in effect, be a bucket handle movement of conjunctiva anteriorly with closure at the limbus and closure posteriorly.

Nonresolving Internal Ostial Obstruction

Vitreous is a possible source of obstruction to the internal ostium and can be difficult to identify. If there is some concern, wiping a dry stroll over the surface of the open sclerostomy will be helpful. Performing a vitrectomy through the sclerostomy can be technically difficult and may need further extension and opening of the space. Removal of lens fragment or iris may be necessary.

Inadequate Follow-up

If the revision is reasonably close in time to the original operation, then there may be significant postoperative inflammation resulting in early fibrosis and impervious scar formation. Follow-up needs to be frequent with an expectation of scarring earlier. If the revision is a number of years from the original operation, then paradoxically the postoperative course may be quite benign. In all cases, close observation for evidence of active scarring requiring early intervention is recommended.

Surgical Endurance and Patient Expectations

With the best surgical intervention there will be failures, disappointments and the need for reintervention. The likelihood for this needs to be telegraphed to the patient and the issues reiterated in the course of treatment. Glaucoma is a disease for life and the relationship of the glaucoma doctor should be a strong one. Consideration of the possible complications is needed for every intervention, but this needs to be carefully weighed against the likely decline in function without them.

Wrong Operation

Uveitis, iridocorneal endothelial syndrome and trauma are particularly high risk for failure of primary filtration surgery and are high risk for failure of revision as well. These eyes may need to have supplemental inflow procedures, such as cyclophotocoagulation, which can reduce the demands on the outflow procedure. Cyclodestructive procedures need to be used with great care in eyes with precarious aqueous production, such as uveitic glaucoma, where ciliary production may be already be poor.
